# Dual-phase machine learning framework for paediatric ventilator liberation: forecasting extubation readiness and nowcasting extubation outcomes

**DOI:** 10.1136/bmjdhai-2025-000138

**Published:** 2025-12-03

**Authors:** Yuxuan Liu, Sofia Cuevas-Asturias, Emma C Alexander, Jake Ormond, Tuan Chen Aw, Angela Aramburo, Samiran Ray, A Aldo Faisal, Padmanabhan Ramnarayan

**Affiliations:** 1 Department of Computing, Imperial College London, London, England, UK; 2 Department of Surgery and Cancer, Imperial College London, London, UK; 3 Southampton Children’s Hospital, Southampton, UK; 4 Paediatric Intensive Care Unit, Imperial College Healthcare NHS Trust, London, UK; 5 Department of Bioengineering, Imperial College London, London, UK; 6 Department of Anaesthesia and Critical Care, Royal Brompton Hospital, London, UK; 7 Paediatric Intensive Care Unit, Great Ormond Street Hospital for Children, London, England, UK; 8 Institute of Artificial and Human Intelligence, University of Bayreuth, Bayreuth, Germany

**Keywords:** Machine Learning, Decision Support Systems, Clinical, Electronic Health Records, Decision Making, Computer-Assisted, Critical Care Outcomes

## Abstract

**Objective:**

To develop and validate a machine learning framework for predicting optimal extubation timing in paediatric intensive care units (PICUs), addressing the clinical need for continuous monitoring of weaning readiness and extubation safety.

**Methods and analysis:**

We developed a dual-phase machine learning framework using data from 3815 ventilation episodes across two UK PICUs. The framework includes (1) a *forecasting* model that predicts extubation readiness up to 12 hours in advance, and (2) a *nowcasting* model that assesses immediate extubation failure risk. Both models used hourly time-series data and were trained using Gated Recurrent Units (GRUs) to capture temporal patterns. Area under the receiver operating characteristic curve (AUROC) and area under the precision-recall curve were used for evaluation on a held-out test set.

**Results:**

The forecasting model achieved an AUROC of 0.85 (95% CI 0.845 to 0.855) and the nowcasting model reached 0.77 (95% CI 0.760 to 0.780). Forecasting performance benefited from longer observation windows (up to 24 hours) and incorporated broader clinical factors (medications, patient characteristics), while nowcasting relied primarily on immediate ventilation parameters. The temporal evolution of risk scores differed markedly between the two models, with the nowcasting model erroneously signalling early readiness.

**Conclusion:**

Our findings demonstrate that distinct modelling strategies are needed to support different stages of ventilator liberation. The proposed dual-phase framework reflects the sequential nature of clinical decision-making and enables structured monitoring throughout the ventilator liberation process.

This study represents the largest machine learning investigation of PICU ventilator liberation to date and demonstrates the feasibility of artificial intelligence-assisted extubation timing in paediatric critical care.

**WHAT IS ALREADY KNOWN ON THIS TOPIC**

- Extubation timing remains challenging in paediatric intensive care, with failure rates of 5–20% and associated mortality risks. Existing prediction models focus on adults and mainly on single-time point risk assessment rather than the sequential nature of clinical decision-making.

**WHAT THIS STUDY ADDS**

- We developed the first dual-phase machine learning framework for paediatric extubation that separately assesses weaning readiness (12 hours ahead) and predicts immediate extubation failure, using the largest paediatric intensive care data set to date (3815 episodes).

**HOW THIS STUDY MIGHT AFFECT RESEARCH, PRACTICE OR POLICY**

- This framework provides a structured approach aligned with clinical workflow that could support timelier and safer ventilator liberation decisions, requiring prospective validation before clinical implementation.

## Introduction

Determining the optimal timing for ventilator liberation is a critical challenge in intensive care, where premature or delayed extubation can lead to serious complications including ventilator-associated pneumonia, delirium, muscle atrophy and 25–50% increased mortality rate.^1–4^ While machine learning has shown promise in predicting extubation outcomes in adult intensive care units (ICUs)^5–7^ and, to a lesser extent, in neonates,^8 9^ paediatric ICUs (PICUs) have received comparatively limited attention. This gap is concerning given that 35–60% of children admitted to PICUs require invasive mechanical ventilation (IMV),^10^ and the paediatric population spans diverse developmental stages with varying pathophysiology, requiring more tailored decision support.

Beyond this population-level gap, most existing studies rely on single-time point prediction models that assess risk only after clinicians decide to extubate.^5^ While offering insight into post-decision outcomes, these models fail to reflect the actual clinical workflow, which involves a sequence of evaluations leading up to extubation. Recent work in adult ICUs has begun to address this limitation through multiphase prediction frameworks that align better with real-world decision-making. These frameworks typically distinguish between two key stages: determining when a patient is ready to begin weaning and evaluating readiness for extubation after support has been reduced. For example, Liu *et al* and Cheng *et al* use ventilation mode transitions to mark weaning onset,^11 12^ while Sheikhalishahi *et al* track positive end-expiratory pressure (PEEP) reductions as readiness signals.^13^

Paediatric ventilation management could benefit from similarly multiphase prediction frameworks. However, PICU application presents unique challenges: many children are extubated directly from hybrid ventilation modes, often without consistent or protocolised transitions.^14 15^ This lack of clear breakpoints complicates the identification of distinct weaning phases and limits the direct applicability of adult-derived approaches.

In this study, we extend the multiphase prediction paradigm to paediatrics by proposing clinically grounded definitions of weaning onset and extubation readiness. Leveraging what is, to our knowledge, the largest retrospective PICU ventilation data set to date, we develop a dual-phase framework that provides complementary predictions aligned with key stages of the extubation process. Specifically, a *forecasting model* first identifies emerging opportunities to initiate extubation readiness testing and a spontaneous breathing trial (SBT), followed by a *nowcasting model* that estimates the likelihood of extubation success immediately before the procedure. This sequential design accounts for the clinical realities of paediatric care while addressing the modelling limitations inherent to retrospective data, offering a practical informatics solution to support timely and individualised ventilator liberation decisions.

## Methods

This study was designed and is reported in concordance with Transparent Reporting of a multivariable prediction model for Individual Prognosis Or Diagnosis - Artificial Intelligence (TRIPOD-AI) guidelines for clinical prediction models using machine learning methods.^16^

### Patient and public involvement

No patient or public involvement was undertaken in this research.

### Task formulation

Ventilator liberation represents a continuum of clinical decisions rather than a discrete event. In practice, clinicians approach this process as two sequential questions: when to initiate reducing ventilator support, and when to remove the endotracheal tube.

Modelling these decisions from retrospective data presents fundamental challenges. First, ground truth labels are asymmetric—we only observe successful extubations, never the counterfactual ‘true readiness’ for non-attempted time points. This creates label bias, as we cannot distinguish between ‘not ready for extubation’ and ‘ready but unrecognised or acted on’ in historical data. Second, traditional indicators used to mark weaning initiation in adults (such as transition to supported modes of ventilation) are inconsistently practised in paediatric practice.^14 15^

**Figure 1:**
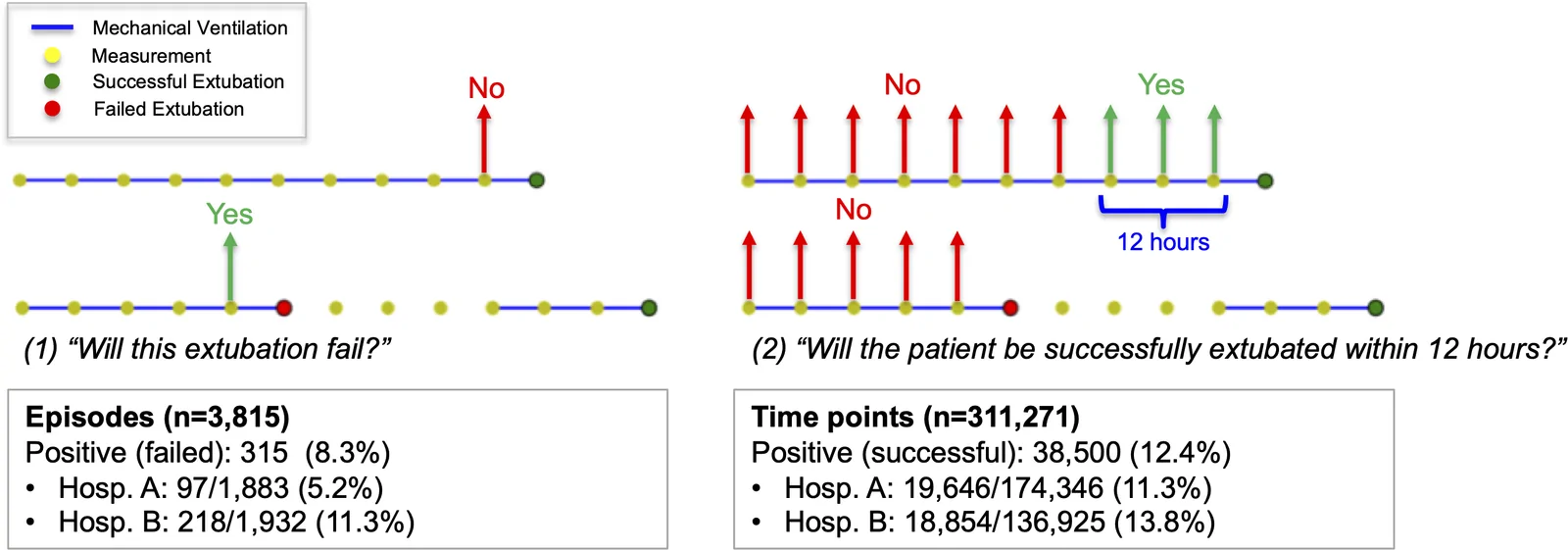
Dual-phase extubation prediction framework.

Addressing these limitations while preserving clinical relevance, we formulated a dual-phase framework with complementary prediction tasks (figure 1):

1. *Forecasting extubation readiness*: predicting successful extubation within the next 12 hours, enabling early identification of emerging weaning windows and triggering extubation readiness testing. This 12-hour horizon aligns with changes in nursing shifts and captures 85.8% of pre-extubation mode transitions in our data set.
2. *Nowcasting extubation outcome*: estimating the risk of extubation failure at the decision point immediately preceding tube removal, mirroring the clinical assessment conducted following an SBT.

Following established paediatric criteria,^17 18^ we defined extubation failure as reintubation within 48 hours postextubation and limited our analysis to first extubation attempts. Both tasks prioritise minority-class prediction to support identification of clinically significant events—optimal weaning windows and potential extubation failures.

### Study data

In this retrospective cohort study, we analysed electronic health record data from mechanically ventilated children (aged 0–17 years) admitted to two London PICUs between January 2013 and December 2022, a general medical-surgical PICU and a mixed cardio-respiratory PICU. Extubation practices in our study PICUs were standardised by use of an extubation readiness checklist and SBT in a large proportion of patients, although some clinical variability remained, for example, in the level of pressure support (PS) set for different ages. We defined complete ventilation episodes as periods from initiation of endotracheal tube ventilation (or from admission if already on IMV) until the first extubation attempt.

To maintain clinical relevance and data quality, we excluded incomplete episodes (death or transfer before extubation attempt), episodes where patients died within 48 hours postextubation (representing terminal extubations), very short episodes (≤12 hours) that typically represent elective postprocedural cases and prolonged episodes (>28 days) involving chronic respiratory failure.^19^ We restricted our analysis to episodes using pressure control (PC) and PS ventilation modes to ensure consistent feature availability. These represent the most used modes in paediatric intensive care, accounting for 76% of IMV in a recent European registry^20^ and 85% of episodes in our cohort. The final cohort included 3815 ventilation episodes.

Further details on inclusion/exclusion criteria and data processing are provided in the online supplemental materials eFigure 1.

10.1136/bmjdhai-2025-000138.supp1 Supplementary data

### Data extraction, preprocessing and features of interest

Data extracted from EHRs included patient demographics (age, sex, weight, diagnostic group, severity of illness at admission using Paediatric Index of Mortality-3 score); vital signs, airway status and ventilator readings charted hourly; blood gas parameters; laboratory blood tests; medications (sedative and neuromuscular blockade infusions, furosemide, vasopressors, corticosteroids) and discharge outcomes (mortality, length of PICU stay). A total of 37 variables were considered features of interest, as described in online supplemental eTable 1.

All features were examined for outliers and errors using Tukey’s method with frequency histograms and univariate statistical methods.^21^ Where feasible, errors were rectified, such as converting tidal volume measurements from litres to millilitres. In instances where correction was impossible, values were designated as missing for subsequent imputation. Additionally, all parameters were capped at clinically plausible limits.^15^ To address missing or irregularly sampled data, we employed a time-limited, parameter-specific sample-and-hold approach, aligning with common practices in health data time series analyses and intuitively mirroring the decision-making process of clinicians.^22^ For the remaining missing data, we used multivariable nearest-neighbour imputation, as our predictors required complete cases.^23^

### Empirical protocols and prediction models

For both tasks, we developed predictive models focusing on temporal analysis of patient data over time. Our methodology acknowledges the dynamic nature of patient recovery trajectory and extubation readiness assessment, considering not only their current state, but also the condition progression over time. To address this, we implemented Gated Recurrent Unit (GRU), a recurrent neural network variant, which has demonstrated superior performance in analysing sequential medical data across various clinical applications.^24^ GRUs are designed to retain information across time and capture evolving physiological patterns, making them well-suited for modelling patient trajectories in a high-resolution ICU setting.

Variables were standardised appropriately based on their distributions, with categorical variables binarised and centred around zero. To determine how much historical data would optimise prediction accuracy, we evaluated model performance using different observation windows, ranging from 1 hour to 48 hours of patient data prior to each prediction time point. To handle class imbalance in our outcome data (figure 1), we used focal loss,^25^ helping models pay extra attention to the rarer but clinically important cases.

For evaluation, we allocated 20% of episodes for testing, with the remaining data divided into training (80%) and validation (20%) data sets. Data splitting was performed at the episode level to ensure no patient appeared in multiple splits and prevent temporal data leakage between training and testing sets. Model performance was evaluated on the test data set using the area under the receiver operating characteristic curve (AUROC) and the area under the precision-recall curve (AUPRC). To ensure robustness, evaluation was repeated five times with different random data splits, and the average performance was reported.

To provide comparable extubation readiness assessment, we integrated both predictions into a unified risk scoring framework. The nowcasting model generates a score (0–1) for immediate extubation failure risk, while the forecasting model produces a score (0–1) indicating the probability of successful extubation within 12 hours. We converted the success probability to a risk metric by interpreting lower success probabilities as higher risk. This integration enables us to monitor the evolution of both immediate and prospective risks over time approaching the actual extubation events.

To understand which clinical factors influence predictions, we computed feature importance using integrated gradients,^26^ a model-agnostic attribution method that assigns importance by measuring each feature’s contribution to the model’s predictions. For GRU-processed sequential features, scores were averaged as absolute values across time to prevent offsetting positive and negative contributions at different time points.^27^ Scores for each model were then normalised relative to their respective maximum values to facilitate fair comparisons.

## Results

Our study included 3815 complete ventilation episodes from 8503 PICU admissions (detailed in online supplemental eFigure 1). Among excluded ventilated cases, data quality exclusions accounted for 768 episodes, including those with missing vital signs >30% of timestamps (n=69) and instances where intubation/extubation time points could not be clearly classified (n=699). Incomplete clinical trajectories comprised episodes ending in transfer/death before extubation attempt (n=440) or mortality within 48 hours of extubation (n=31).

In the study cohort (n=3815 episodes), the median age was 8.0 months (IQR 2.0–34.0), with cardiovascular (43.7%) and respiratory (29.5%) conditions as the predominant diagnoses. The median IMV duration was 66.0 hours (IQR 29.0–118.0) (table 1). In the nowcasting model, 3500 (91.7%) attempted extubations were successful, while 315 (8.3%) required reintubation within 48 hours. Failed extubations occurred in younger children (median age 5.0 vs 8.0 months, p<0.001) and were associated with significantly higher rates of neuromuscular blockade use during IMV (7.0% vs 0.2%, p<0.001). Children experiencing extubation failure were more likely to be on PC ventilation in the hour preceding extubation rather than PS (65.4% vs 35.9%, p<0.001) and required higher respiratory support as indicated by mean airway pressure (8.0 vs 7.0 cm H₂O, p<0.001). For the forecasting task, 42 000 (14.5%) of 290 156 analysed time points were classified as ‘ready-to-wean’ based on successful extubation within the next 12 hours. These ready states were associated with more favourable physiological parameters, including lower average heart rates (123 vs 127 bpm, p<0.001) and better ventilation with lower partial pressure of carbon dioxide (5.90 vs 6.14 kPa, p<0.001) (detailed in online supplemental eTable 2).

**Table 1:** Demographic and clinical characteristics of study cohort

	Overall
Unique ventilation episodes (N)	3815
Age, months (median, IQR)	8.0 (2.0–34.0)
Male sex (N, %)	2145 (56.2)
Ethnicity (N, %)	
White or white British	1693 (44.4)
Black or black British	500 (13.1)
Asian or Asian British	690 (18.1)
Others (including missing)	932 (24.4)
Primary diagnosis (N, %)	
Cardiovascular	1667 (43.7)
Respiratory	1124 (29.5)
Neurological	406 (10.6)
Gastrointestinal	56 (1.5)
Infection	160 (4.2)
Others	402 (10.5)
PIM-3 predicted probability of death (median, IQR)	0.020 (0.008–0.053)
Admitted with mechanical ventilation (N, %)	3022 (70.3)
Length of ventilation episode, hours (median, IQR)	66.0 (29.0–118.0)

PIM-3, Paediatric Index of Mortality 3

**Figure 2:**
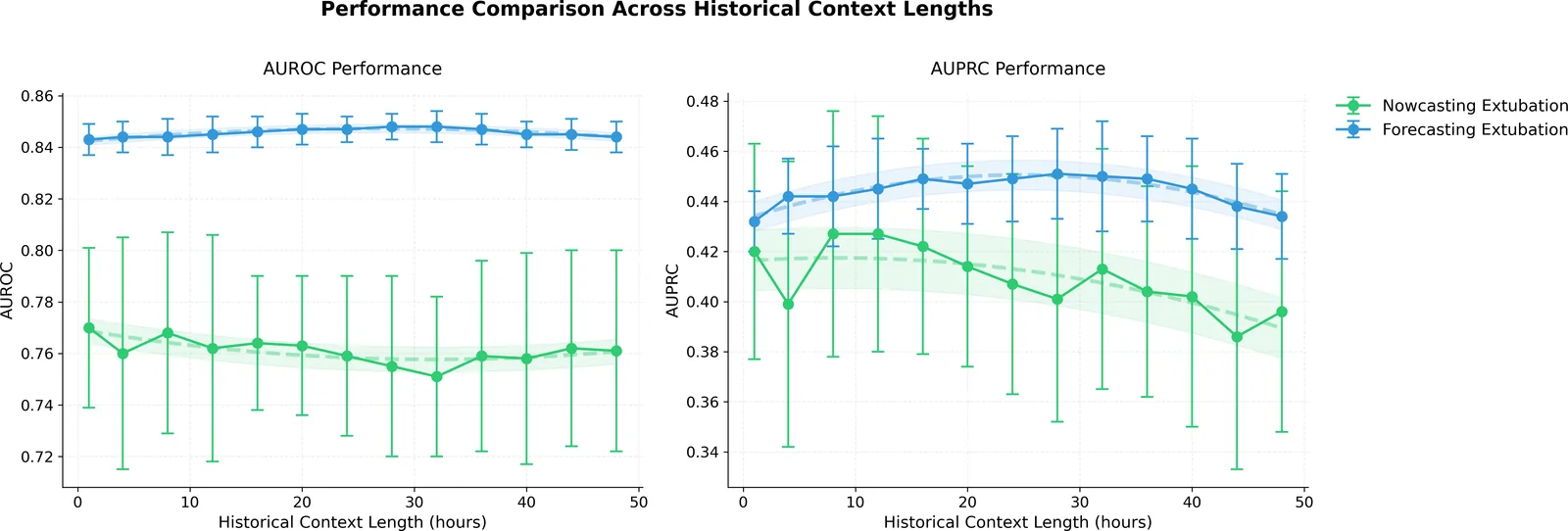
Model performance across different historical context lengths. AUPRC, area under the precision-recall curve; AUROC, area under the receiver operating characteristic curve.

The final cohort was randomly split into development (n=3052), evaluation (n=611) and testing (n=763) data sets. The optimised nowcasting model reached an AUROC of 0.770 (95% CI 0.760 to 0.780) and AUPRC of 0.420 (95% CI 0.410 to 0.430), while the forecasting model achieved an AUROC of 0.850 (95% CI 0.845 to 0.855) and AUPRC of 0.451 (95% CI 0.446 to 0.456) (figure 2). To assess the impact of historical data on prediction performance, we further evaluated models with varying lengths of clinical history from 1 to 48 hours. The nowcasting task demonstrated consistent decline in performance with increasing historical context (AUROC decreasing from 0.770 to 0.760, AUPRC from 0.420 to 0.396) and exhibited wider CIs. In contrast, the forecasting task showed initial improvement with increasing historical context up to 24 hours (AUROC from 0.845 to 0.850, AUPRC from 0.432 to 0.451), followed by gradual decline in AUPRC, while maintaining stable AUROC. The model exhibited narrow CIs throughout. These patterns indicate different optimal historical context requirements for the two prediction tasks.

**Figure 3:**
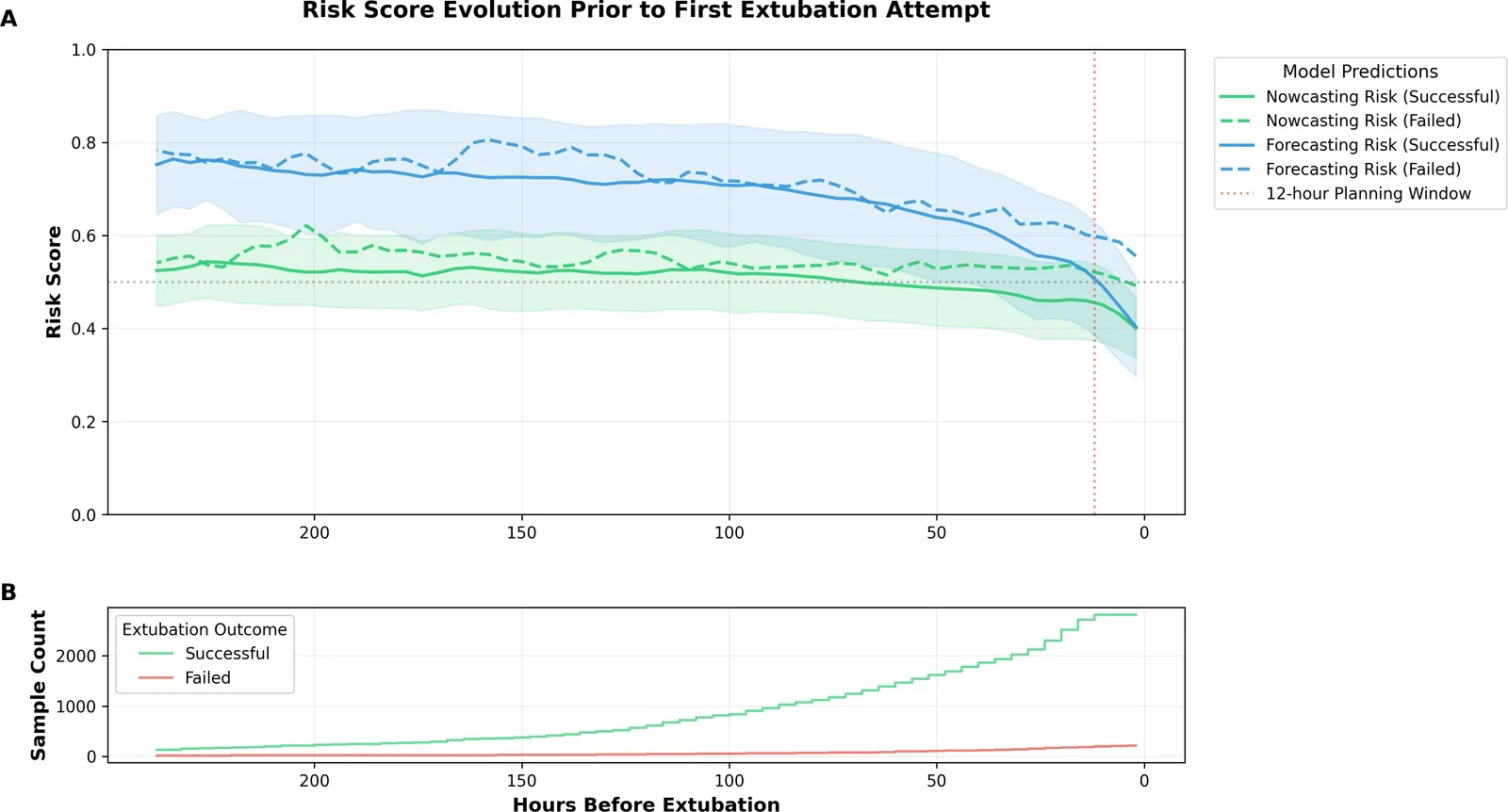
Risk score evolution and sample distribution prior to extubation.

The temporal evolution of risk scores preceding extubation exhibited distinct patterns between successful and failed cases, as illustrated in figure 3. For successful extubations, both the nowcasting risk score (green solid line) and the forecasting risk score (blue solid line) demonstrated a descending trajectory. Notably, the forecasting risk score accurately intersected the 0.5 decision threshold at the intended 12-hour planning window, indicating optimal timing prediction. In contrast, the nowcasting risk score incorrectly traversed this boundary earlier, at approximately 70 hours pre-extubation, demonstrating temporal generalisation failure when applied outside its intended time context. Failed extubation cases (dashed lines) maintained consistently elevated risk scores in both tasks and displayed less improvement approaching extubation. The discrepancy between successful and failed cases was particularly evident in the forecasting risk assessment, with separation increasing as extubation approached, whereas the nowcasting risk scores showed more overlap and earlier boundary crossing than clinically appropriate.

**Figure 4:**
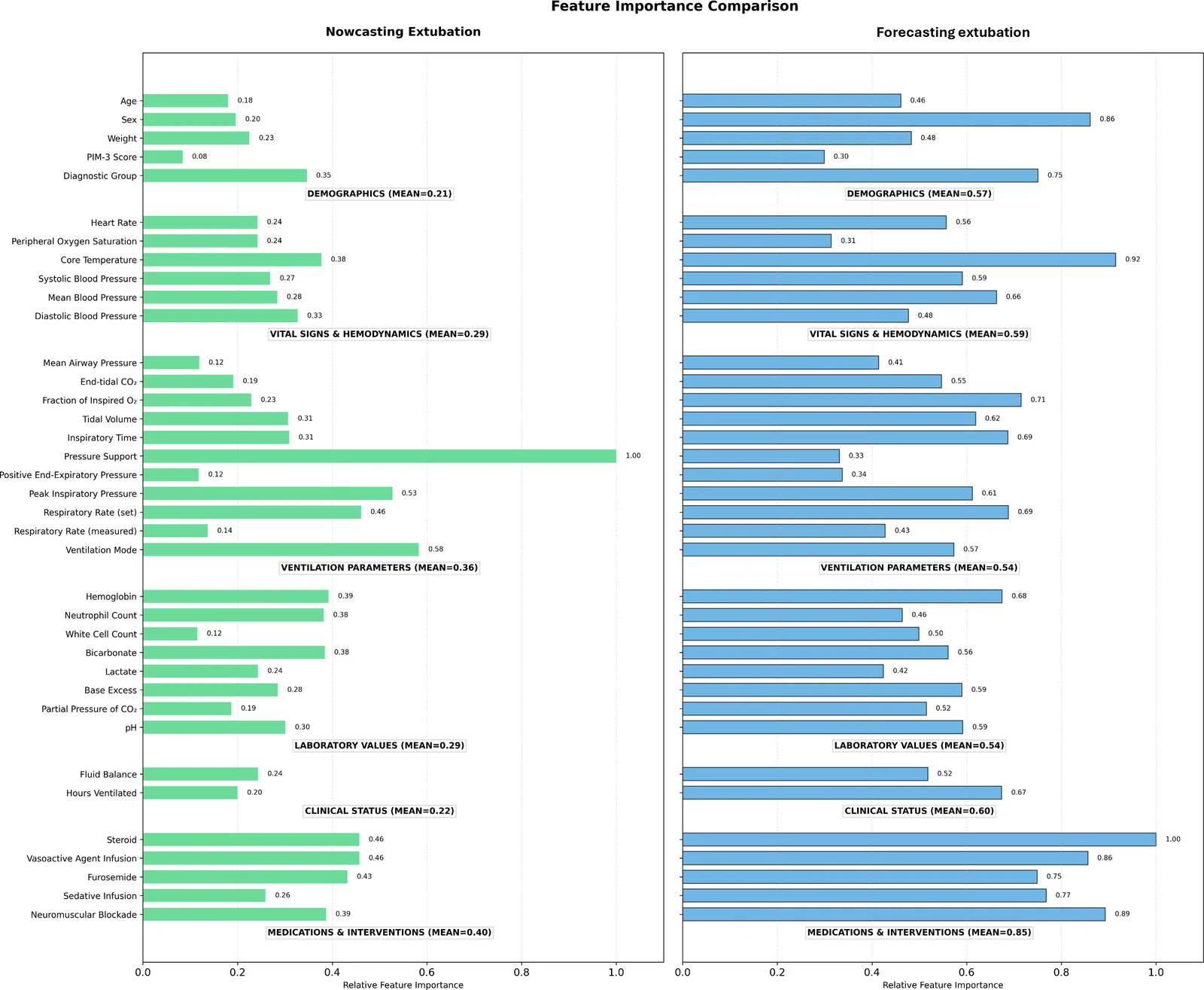
Feature importance comparison between dual-phase models. PIM-3, Paediatric Index of Mortality 3.

Feature importance analysis revealed distinct patterns between the two tasks (figure 4). The nowcasting model relied mostly on ventilation parameters, with PS level (in cmH_2_O) showing maximum importance, followed by ventilation mode and peak inspiratory pressure. In contrast, the forecasting model demonstrated more distributed importance across multiple clinical domains, with consistently high importance across medication interventions, physiological parameters and demographic characteristics. Notably, while ventilation parameters dominated immediate extubation outcome prediction, their relative importance was substantially lower in forecasting—patient factors like sex (relative importance 0.86) and diagnostic group (relative importance 0.75) gained prominence in determining optimal weaning timing.

## Discussion

In this large cohort study of 3815 paediatric mechanical ventilation episodes, we developed a dual-phase prediction framework that addresses the challenge of determining optimal extubation timing. Our approach mirrors the sequential nature of clinical ventilator liberation by distinguishing between forecasting future extubation readiness (proxying weaning initiation) and nowcasting immediate extubation outcomes (similar to traditional extubation failure prediction).

Our framework adapts concepts validated in adult critical care to a paediatric context. To our knowledge, this is the largest machine learning study of extubation decision support in PICUs to date.

Our models achieved strong predictive performance, with the nowcasting model reaching an AUROC of 0.77 and the forecasting model achieving an AUROC of 0.85. While these metrics fall within ranges reported in previous extubation prediction studies (AUROC 0.75–0.85),^1^ direct comparisons are limited by population differences.

Our analysis of temporal risk score evolution confirmed the need for a multiphase approach and highlighted the continuous nature of the extubation process. Notably, the nowcasting model’s risk score crossed the decision threshold prematurely—suggesting likely success nearly 70 hours before extubation—when applied to earlier time points, demonstrating that models designed for immediate extubation assessment cannot be reliably generalised for early readiness detection. This temporal generalisation failure represents an important but under-recognised methodological pitfall in extubation prediction research, where single-time point models may be implicitly applied across multiple time contexts without specific validation. This finding validates our framework’s structure: systematic monitoring for weaning readiness, followed by targeted assessment of immediate extubation risk once readiness indicators emerge.

The differential temporal requirements of our two prediction tasks further support this sequential approach. While the nowcasting model performed best with recent clinical data, showing declining performance with extended history, the forecasting model required longer observation periods (up to 24 hours) to achieve optimal performance. This pattern mirrors clinical reasoning: immediate extubation decisions rely primarily on current physiological status, while identifying optimal timing to start weaning requires consideration of broader clinical trajectory and stability.

Feature importance analysis provided insight into and validation of our models’ clinical relevance, which accounted for complex relationships between individual variables. The nowcasting model relied predominantly on ventilation parameters, particularly PS levels and degree of spontaneous ventilation—factors that clinicians routinely assess immediately before extubation attempts. In contrast, the forecasting model showed more distributed importance across medication interventions, physiological parameters and patient characteristics. This broader consideration aligns with how clinicians assess overall recovery and readiness to begin weaning, where treatment response and patient-specific factors play significant roles alongside respiratory parameters.

The complementary nature of these models provides a more comprehensive framework for ventilation liberation than single-time point prediction. By first identifying potential windows of extubation readiness to prompt extubation readiness testing (forecasting) and then validating the safety of the extubation attempt at the point of decision (nowcasting), this approach shows practical clinical value, achieving strong predictive performance using only routine physiological monitoring data available in standard ICU care.

## Limitations and future directions

Several limitations of our study warrant discussion. First, our choice of a 12-hour prediction window for the forecasting task, though aligned with nursing shifts and capturing most pre-extubation mode transitions, remains somewhat arbitrary. Alternative approaches to defining weaning readiness could be explored, such as the PEEP reduction method used by Sheikhalishahi *et al* in adult critical care.^13^ The optimal timeframe likely varies across patient populations and clinical settings.

Second, retrospective label bias remains a key limitation, as the ground truth for ‘extubation readiness’ is only observed when extubation occurs. Moreover, national ventilator-liberation protocols were standardised in UK PICUs during 2017–2019 as part of the Sedation and Weaning in Children (SANDWICH) clinical trial.^28^ While the underlying readiness criteria remained consistent, this transition could have introduced minor temporal variation in labelling. Future prospective studies, including clinical trials, will be critical to evaluate whether these predictions generalise across populations and over time.

Third, we simplified the clinical problem by excluding complex scenarios such as repeated extubation attempts, prolonged ventilation episodes and cases ending in transfer out or mortality. While necessary for model development and to avoid bias, this limits generalisability to the full spectrum of PICU patients. Our cohort’s extubation failure rate (8.3%) is consistent with established paediatric reports, indicating a representative extubation population. As an exploratory check, we applied the forecasting model to excluded patients who died or were transferred out prior to an extubation attempt. We found that readiness predictions were infrequent (2952 of 37 995 readiness windows across 119 of 394 patients) and generally distant from terminal events, supporting model stability outside its intended population. Similarly, we faced data quality challenges that required algorithmic classification of ventilation status and could not incorporate important clinical factors like secretion management, cough effectiveness, and neurological status due to inconsistent documentation.

Fourth, upper airway obstruction accounts for many cases of extubation failure. Our models used routinely collected physiological data that did not include any measures of the risk for upper airway obstruction (other than duration of prior ventilation), which may have compromised the performance of the models. Future studies integrating real-time clinical assessments could provide enhanced prediction.

Finally, our modelling approach prioritised consistency across tasks rather than optimising each prediction independently. Future work may explore tailored models or alternative architectures better suited to each phase (eg, XGBoost for nowcasting, where temporal dynamics are less critical).

Despite these limitations, our findings support the value of distinct predictive approaches for different stages of ventilator liberation and lay the groundwork for future implementation and validation.

## Conclusions

We present a dual-phase prediction framework that reflects the sequential nature of extubation decision-making in paediatric intensive care. By distinguishing between early identification of weaning readiness and immediate assessment of extubation safety, our approach addresses limitations of single time-point models. This dual perspective supports both proactive resource planning and family communication, as well as point-of-decision safety assessment, thereby covering the entire ventilator liberation process rather than isolated prediction tasks. While further validation is needed, this framework offers a structured, clinically aligned approach to support safer and more timely ventilator liberation in children.

## Data Availability

Data are available upon reasonable request. The data sets generated and/or analysed during the current study are not publicly available due to patient privacy regulations and institutional policies but are available from the corresponding author on reasonable request.

## References

[1] Torrini F, Gendreau S, Morel J, et al. Prediction of extubation outcome in critically ill patients: a systematic review and meta-analysis. Crit Care 2021;25. 10.1186/s13054-021-03802-3 PMC8591441 34782003

[2] Thille AW, Richard JCM, Brochard L. The decision to extubate in the intensive care unit. Am J Respir Crit Care Med 2013;187:1294–302. 10.1164/rccm.201208-1523CI 23641924

[3] Khemani RG, Sekayan T, Hotz J, et al. Risk Factors for Pediatric Extubation Failure: The Importance of Respiratory Muscle Strength. Crit Care Med 2017;45:e798–805. 10.1097/CCM.0000000000002433 28437378 PMC5511064

[4] Miura S, Butt W, Thompson J, et al. Recurrent Extubation Failure Following Neonatal Cardiac Surgery Is Associated with Increased Mortality. Pediatr Cardiol 2021;42:1149–56. 10.1007/s00246-021-02593-2 33864485 PMC8052939

[5] Kwong MT, Colopy GW, Weber AM, et al. The efficacy and effectiveness of machine learning for weaning in mechanically ventilated patients at the intensive care unit: a systematic review. Bio-Des Manuf 2019;2:31–40. 10.1007/s42242-018-0030-1

[6] Chen T, Xu J, Ying H, et al. Prediction of Extubation Failure for Intensive Care Unit Patients Using Light Gradient Boosting Machine. IEEE Access 2019;7:150960–8. 10.1109/ACCESS.2019.2946980

[7] Fabregat A, Magret M, Ferré JA, et al. A Machine Learning decision-making tool for extubation in Intensive Care Unit patients. Comput Methods Programs Biomed 2021;200:105869. 10.1016/j.cmpb.2020.105869 33250280

[8] Natarajan A, Lam G, Liu J, et al. Prediction of extubation failure among low birthweight neonates using machine learning. J Perinatol 2023;43:209–14. 10.1038/S41372-022-01591-3;SUBJMETA=1720,174,1785,308,692,699,700;KWRD=EPIDEMIOLOGY,PAEDIATRICS,RESPIRATORY+TRACT+DISEASES 36611107 PMC10348822

[9] Tao Y, Ding X, Guo WL. Using machine-learning models to predict extubation failure in neonates with bronchopulmonary dysplasia. BMC Pulm Med 2024;24:308. 10.1186/s12890-024-03133-3 38956528 PMC11218173

[10] PICANet state of the nation report 2022 – picanet. 2025. Available: https://www.picanet.org.uk/2023/03/09/picanet-state-of-the-nation-report-2022/

[11] Liu C-F, Hung C-M, Ko S-C, et al. An artificial intelligence system to predict the optimal timing for mechanical ventilation weaning for intensive care unit patients: A two-stage prediction approach. Front Med (Lausanne) 2022;9:935366. 10.3389/fmed.2022.935366 36465940 PMC9715756

[12] Cheng K-H, Tan M-C, Chang Y-J, et al. The Feasibility of a Machine Learning Approach in Predicting Successful Ventilator Mode Shifting for Adult Patients in the Medical Intensive Care Unit. Med Bogota Colomb 2022;58:360. 10.3390/medicina58030360 PMC8949015 35334536

[13] Sheikhalishahi S, Kaspar M, Zaghdoudi S, et al. Predicting Successful Weaning from Mechanical Ventilation by Reduction in Positive End-expiratory Pressure Level Using Machine Learning. PLOS Digit Health 2024;3:e0000478. 10.1371/journal.pdig.0000478 38536802 PMC10971612

[14] Newth CJL, Venkataraman S, Willson DF, et al. Weaning and extubation readiness in pediatric patients. Pediatr Crit Care Med 2009;10:1–11. 10.1097/PCC.0b013e318193724d 19057432 PMC2849975

[15] Abu-Sultaneh S, Iyer NP, Fernández A, et al. Executive Summary: International Clinical Practice Guidelines for Pediatric Ventilator Liberation, A Pediatric Acute Lung Injury and Sepsis Investigators (PALISI) Network Document. Am J Respir Crit Care Med 2023;207:17–28. 10.1164/rccm.202204-0795SO 36583619 PMC9952867

[16] Collins GS, Moons KGM, Dhiman P, et al. TRIPOD+AI statement: updated guidance for reporting clinical prediction models that use regression or machine learning methods. BMJ 2024;385:e078378. 10.1136/bmj-2023-078378 38626948 PMC11019967

[17] Ramnarayan P, Richards-Belle A, Drikite L, et al. Effect of High-Flow Nasal Cannula Therapy vs Continuous Positive Airway Pressure Following Extubation on Liberation From Respiratory Support in Critically Ill Children. JAMA 2022;327:1555. 10.1001/jama.2022.3367 35390113 PMC8990361

[18] Baisch SD, Wheeler WB, Kurachek SC, et al. Extubation failure in pediatric intensive care incidence and outcomes. Pediatr Crit Care Med 2005;6:312–8. 10.1097/01.PCC.0000161119.05076.91 15857531

[19] Kawaguchi A, Fernandez A, Baudin F, et al. Longventkids study: a prospective cohort study on prolonged mechanical ventilated children. SSRN [Preprint] 2024. 10.2139/ssrn.4847208

[20] van Vliet R, van Meenen DMP, Blokpoel RGT, et al. Ventilator Settings in Critically Ill Pediatric Patients (VESPer)—insights from a European Registry. Intensive Care Med – Paediatr Neonatal 2025;3:1–9. 10.1007/s44253-025-00069-2

[21] Keselman HJ, Rogan JC. The Tukey multiple comparison test: 1953–1976. Psychol Bull 1977;84:1050–6. 10.1037/0033-2909.84.5.1050

[22] MAS caleb wayne by, uthor a, orlando tp. detecting hazardous intensive care patient episodes using real-time mortality models. 2009.

[23] Tutz G, Ramzan S. Improved methods for the imputation of missing data by nearest neighbor methods. Computational Statistics & Data Analysis 2015;90:84–99. 10.1016/j.csda.2015.04.009

[24] Cho K, van Merrienboer B, Gulcehre C, et al. Learning phrase representations using rnn encoder–decoder for statistical machine translation. Proceedings of the 2014 Conference on Empirical Methods in Natural Language Processing (EMNLP); Stroudsburg, PA, USA, 2014;Doha, Qatar. 10.3115/v1/D14-1179 Available: http://aclweb.org/anthology/D14-1

[25] Lin T-Y, Goyal P, Girshick R, et al. Focal Loss for Dense Object Detection. IEEE Trans Pattern Anal Mach Intell 2017;42:318–27. 10.1109/TPAMI.2018.2858826 30040631

[26] Sundararajan M, Taly A, Yan Q. Axiomatic attribution for deep networks. 34th International Conference on Machine Learning, ICML; 2017:5109–18.

[27] Ismail AA, Gunady M, Bravo HC, et al. Benchmarking Deep Learning Interpretability in Time Series Predictions. Adv Neural Inf Process Syst 2020.

[28] Blackwood B, Tume LN, Morris KP, et al. Effect of a Sedation and Ventilator Liberation Protocol vs Usual Care on Duration of Invasive Mechanical Ventilation in Pediatric Intensive Care Units: A Randomized Clinical Trial. JAMA 2021;326:401–10. 10.1001/jama.2021.10296 34342620 PMC8335576

