# Supplementary material for: Dual-phase machine learning framework for paediatric ventilator liberation: forecasting extubation readiness and nowcasting extubation outcomes
Source: BMJ Digit Health Ai. 2025 Dec 3;1(1):e000138. doi: 10.1136/bmjdhai-2025-000138 (PMC13492549; doi:10.1136/bmjdhai-2025-000138)
Supplement: Supplementary file 1 [file bmjdhai-1-1-bmjdhai-2025-000138-s001.pdf]

Supplemental Information

Liu et al. A Dual-Phase Machine Learning Framework for Pediatric Ventilator Liberation: Forecasting Extubation Readiness and Nowcasting Extubation Outcomes

This supplemental material has been provided by the authors to give readers additional information about their work.

Contents:

- eFigure 1. Study Flow Diagram of Patient Selection and Data Split
- eTable 1. Features of Interest Used in Prediction Models
- eTable 2. Class-wise Comparison of Clinical Characteristics in Two Prediction Tasks
- Additional Methods Details

eFigure 1. Study Flow Diagram of Patient Selection and Data Split

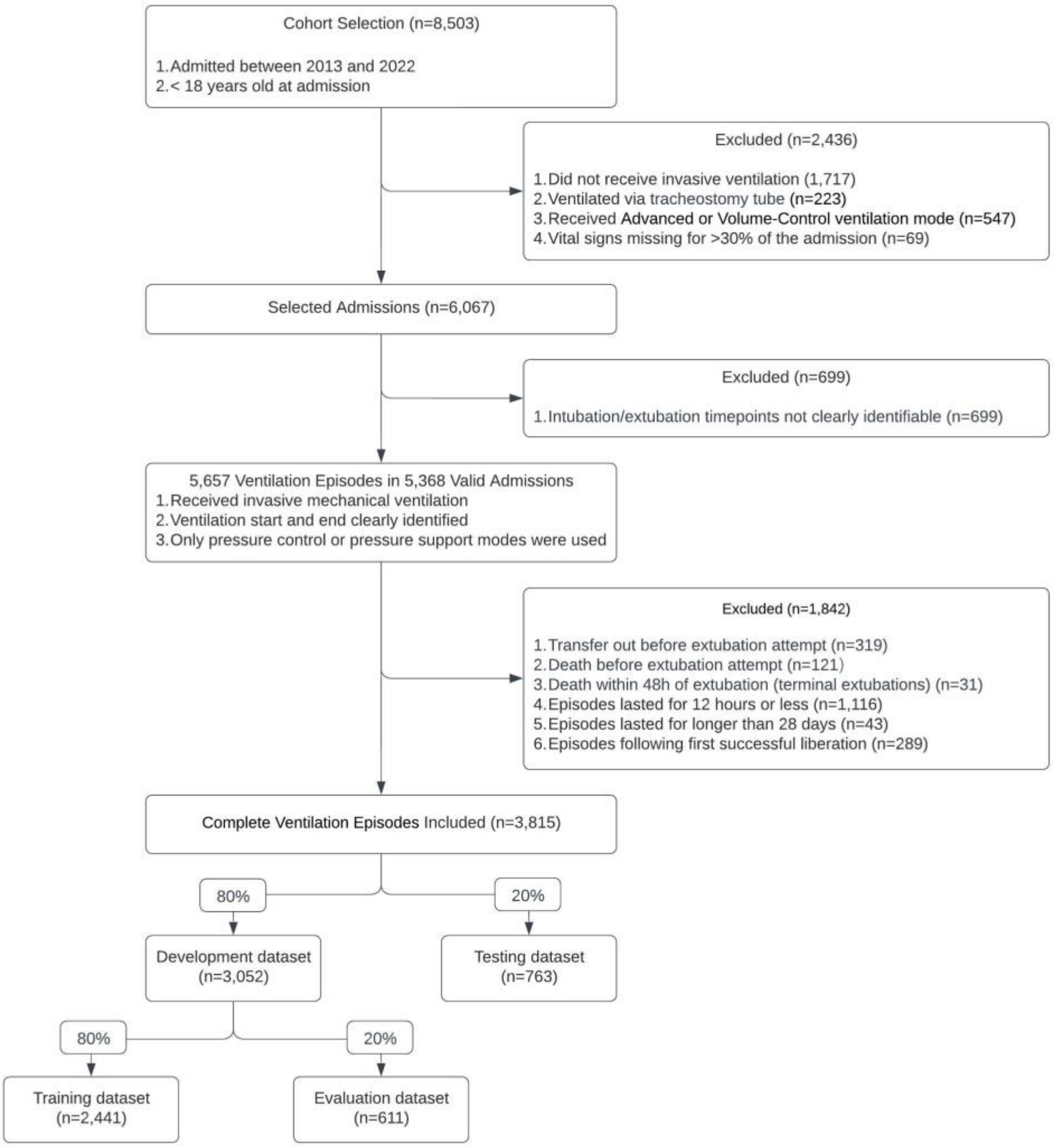

Flow diagram showing patient selection, exclusion criteria, and data partitioning strategy for model development. From 8,503 initial admissions, 3,815 complete ventilation episodes were included after applying exclusion criteria. The final cohort was partitioned into development and testing sets, with further subdivision of the development set for model training and evaluation.

eTable 1. Features of Interest Used in Prediction Models

**Demographics and outcomes**	**Hourly vital signs and ventilator data**
Age	Heart rate (bpm)
Sex	Systolic blood pressure (mm Hg)
Weight (kg)	Mean blood pressure (mm Hg)
Diagnostic group	Diastolic blood pressure (mm Hg)
PIM-3 score	Core temperature (C)
**Blood gases and laboratory tests**	Mean airway pressure (cm H2O)
pH	Measured respiratory rate
pCO2 (kPa)	End tidal CO2 (kPa)
Base excess (mmol/L)	Peripheral oxygen saturation
Lactate (mmol/L)	Tidal volume (ml/kg)
Bicarbonate (mmol/L)	Hours on ventilator
White cell count	Ventilator mode†
Neutrophil count	Fraction of inspired oxygen
Haemoglobin (g/L)	Peak inspiratory pressure
**Medications (yes/no)**	Positive end-expiratory pressure
Sedative infusion	Pressure Support (cm H2O)
Neuromuscular blockade	Set respiratory rate
Vasoactive agent infusion	Inspiratory time (seconds)
Furosemide	Net fluid balance (ml)
Corticosteroids	

Complete list of 37 variables considered as features of interest, including patient demographics, vital signs, ventilator parameters, blood gas values, laboratory tests, medications, and discharge outcomes.

† Two ventilation modes were included in the analysis: (1) Pressure control (PC): Pressure control-biphasic positive airway pressure (PC-BIPAP), which aligns with pressure control intermittent mandatory ventilation with set-point targeting scheme (PC-IMVs) in international ventilation taxonomy; (2) Pressure Support (PS): Spontaneous continuous positive airway pressure with pressure support (SPN-CPAP PS), which aligns with pressure control continuous spontaneous ventilation (PC-CSV) in international ventilation taxonomy.

eTable 2. Class-wise Comparison of Clinical Characteristics in Two Prediction Tasks

Features	Extubation Success (n=3500)	Extubation Failure (n=315)	p-value	Effect Size	Not Ready-to-Wean (n=248156)	Ready-to-Wean (n=42000)	p-value	Effect Size
Ventilator Hours	65.00 (30.00 - 113.00)	55.00 (25.50 - 108.00)	0.024	-0.076	137.00 (88.00 - 233.00)	65.00 (30.00 - 113.00)	<0.001	-0.527
Neuromuscular blockade	8 (0.2%)	22 (7.0%)	<0.001	N/A	18053 (7.3%)	476 (1.1%)	<0.001	N/A
Sedative infusion	1252 (35.8%)	152 (48.3%)	<0.001	N/A	119380 (48.1%)	20859 (49.7%)	<0.001	N/A
Furosemide	395 (11.3%)	39 (12.4%)	0.621	N/A	30021 (12.1%)	5037 (12.0%)	0.548	N/A
Vasoactive agent infusion	836 (23.9%)	96 (30.5%)	0.011	N/A	59108 (23.8%)	10358 (24.7%)	<0.001	N/A
Corticosteroids	247 (7.1%)	15 (4.8%)	0.154	N/A	1353 (0.5%)	1251 (3.0%)	<0.001	N/A
Ventilator mode (PC)	1258 (35.9%)	206 (65.4%)	<0.001	N/A	233311 (94.0%)	27672 (65.9%)	<0.001	N/A
Ventilator mode (PS)	2242 (64.1%)	109 (34.6%)	<0.001	N/A	14845 (6.0%)	14328 (34.1%)	<0.001	N/A
Mean airway pressure (cm H2O)	7.00 (6.00 - 8.00)	8.00 (7.00 - 9.00)	<0.001	0.375	9.00 (8.00 - 11.00)	7.00 (7.00 - 8.00)	<0.001	-0.535
Measured respiratory rate	28.00 (21.00 - 38.00)	29.00 (24.00 - 37.00)	0.078	0.060	26.00 (22.00 - 33.00)	26.00 (20.00 - 35.00)	0.242	0.004
End tidal CO2 (kPa)	5.20 (4.50 - 5.90)	5.10 (4.30 - 5.80)	0.005	-0.095	5.20 (4.48 - 6.00)	5.20 (4.60 - 5.90)	0.001	0.010
Core temperature (C)	37.20 (36.80 - 37.50)	37.00 (36.55 - 37.50)	<0.001	-0.153	37.00 (36.50 - 37.40)	37.20 (36.80 - 37.50)	<0.001	0.150
Heart rate (bpm)	125.00 (108.00 - 140.00)	132.00 (112.00 - 148.00)	<0.001	0.154	127.00 (110.00 - 143.00)	123.00 (107.00 - 138.00)	<0.001	-0.101
Peripheral oxygen saturation	97.00 (95.00 - 99.00)	97.00 (93.00 - 99.00)	0.122	-0.052	97.00 (94.00 - 99.00)	97.00 (95.00 - 99.00)	<0.001	0.017
Systolic blood pressure (mm Hg)	90.00 (77.00 - 104.00)	82.00 (70.00 - 98.50)	<0.001	-0.195	81.00 (69.00 - 95.00)	86.00 (74.00 - 99.00)	<0.001	0.127
Mean blood pressure (mm Hg)	63.00 (54.00 - 73.00)	57.00 (49.00 - 68.00)	<0.001	-0.182	56.00 (48.00 - 65.00)	58.00 (50.00 - 68.00)	<0.001	0.127
Diastolic blood pressure (mm Hg)	49.00 (41.00 - 58.00)	44.00 (38.00 - 53.50)	<0.001	-0.164	43.00 (36.00 - 51.00)	45.00 (38.00 - 54.00)	<0.001	0.119
pH	7.41 (7.37 - 7.45)	7.39 (7.34 - 7.44)	<0.001	-0.166	7.39 (7.35 - 7.44)	7.41 (7.36 - 7.45)	<0.001	0.109
PCO2 (kPa)	5.89 (5.20 - 6.70)	5.92 (5.18 - 6.78)	0.834	-0.007	6.14 (5.30 - 7.29)	5.90 (5.20 - 6.79)	<0.001	-0.114
Base excess (mmol/L)	3.20 (-0.40 - 7.50)	1.60 (-2.30 - 5.85)	<0.001	-0.156	3.10 (-0.80 - 8.30)	2.90 (-1.00 - 7.60)	<0.001	-0.023
Lactate (mmol/L)	1.00 (0.70 - 1.30)	1.10 (0.80 - 1.60)	<0.001	0.165	1.00 (0.79 - 1.40)	1.00 (0.70 - 1.30)	<0.001	-0.047
Bicarbonate (mmol/L)	27.00 (23.90 - 31.30)	25.60 (22.20 - 29.50)	<0.001	-0.167	27.30 (23.90 - 32.20)	26.80 (23.60 - 31.30)	<0.001	-0.047
White cell count	9.66 (7.20 - 12.80)	10.10 (7.60 - 13.60)	0.066	0.063	9.50 (6.90 - 13.10)	9.70 (7.20 - 12.80)	0.001	0.010
Neutrophil count	5.70 (3.60 - 8.41)	6.19 (3.70 - 9.06)	0.074	0.061	5.50 (3.30 - 8.60)	5.70 (3.60 - 8.50)	<0.001	0.018
Haemoglobin (g/L)	107.00 (94.00 - 122.00)	111.00 (96.00 - 129.00)	0.003	0.100	104.00 (92.00 - 122.00)	107.00 (94.00 - 123.00)	<0.001	0.057
Net fluid balance (ml)	-0.80 (-19.00 - 10.00)	2.30 (-13.95 - 10.75)	0.013	0.084	3.00 (-11.00 - 17.00)	0.70 (-15.00 - 12.80)	<0.001	-0.083
Fraction of inspired oxygen	25.00 (21.00 - 30.00)	30.00 (25.00 - 40.00)	<0.001	0.226	30.00 (25.00 - 40.00)	25.00 (21.00 - 31.00)	<0.001	-0.268
Peak inspiratory pressure	0.00 (0.00 - 15.00)	15.00 (0.00 - 19.00)	<0.001	0.406	18.00 (15.00 - 21.00)	15.00 (0.00 - 16.00)	<0.001	-0.561
Set respiratory rate	0.00 (0.00 - 5.00)	10.00 (0.00 - 22.00)	<0.001	0.431	20.00 (14.00 - 25.00)	5.00 (0.00 - 15.00)	<0.001	-0.555
Positive end-expiratory pressure	5.00 (5.00 - 5.00)	5.00 (5.00 - 5.00)	<0.001	0.129	5.00 (5.00 - 6.00)	5.00 (5.00 - 5.00)	<0.001	-0.268
Inspiratory time (seconds)	0.00 (0.00 - 0.80)	0.70 (0.00 - 0.85)	<0.001	0.263	0.80 (0.70 - 1.00)	0.70 (0.00 - 0.89)	<0.001	-0.243
Pressure support (cm H2O)	5.00 (0.00 - 8.00)	0.00 (0.00 - 5.00)	<0.001	-0.274	0.00 (0.00 - 0.00)	0.00 (0.00 - 5.00)	<0.001	0.281
Tidal volume (ml/kg)	7.22 (5.45 - 9.33)	6.96 (5.13 - 8.93)	0.112	-0.054	7.18 (5.61 - 8.86)	7.29 (5.62 - 9.25)	<0.001	0.036

For the nowcasting extubation outcome prediction task (left columns), characteristics were measured in the hour immediately preceding extubation attempts (n=3,815). For the forecasting extubation readiness prediction task (right columns), characteristics were measured at hourly intervals throughout the ventilation period (n=290,156). For time-varying measurements, values represent the last measurement in the observation window. Continuous variables are presented as median (IQR) and categorical variables as count (percentage). Effect sizes for continuous variables were calculated using Cohen's d, while N/A indicates categorical variables where effect size is not applicable. While the prediction models were trained with varying historical window sizes (1-48 hours) as a hyperparameter, for the purpose of this baseline comparison, we look at only the last measurements to ensure consistent comparison across all cases.

Additional Methods Details

Rationale for Time-Based Prediction Framework

Analysis of our 3,815 pediatric ventilation episodes revealed that 1,464 patients (38.4%) were extubated directly from pressure control (PC) mode without transitioning to pressure support (PS). While extubation from PS was associated with higher success rates (95.4% vs. 85.9%), the substantial proportion of direct PC extubations demonstrates that ventilator mode transitions cannot reliably mark weaning initiation in pediatric practice. Using mode changes as a proxy for weaning onset would exclude over one-third of valid cases, limiting both clinical relevance and model generalizability. This finding motivated our time-based forecasting approach rather than the transition-dependent methods used in adult studies.

Ventilation Status Classification Algorithm

A significant challenge in ICU data analysis is inconsistent recording of ventilation information in electronic health records. Our dataset lacked clinically labeled intubation and extubation times, which precluded direct identification of ventilation episodes. To address this limitation, we developed a multi-evidence algorithm that classified ventilation status hourly by cross-referencing three sources of clinical information:

1. **Airway documentation**: Keyword detection for invasive airways ('tube', 'ett') while excluding nasal airways
2. **Ventilation mode classification**: Categorization as invasive (PC, PS) versus non-invasive (NIV, high-flow, no support)
3. **Ventilation parameter presence**: Simultaneous documentation of end tidal CO_2, PEEP settings, and elevated FiO_2

Resolution logic: Single evidence source was accepted as definitive; two sources were accepted if concordant or flagged as indeterminate if conflicting; three sources were resolved by majority consensus or flagged as indeterminate if all disagreed. Episodes with >30% indeterminate timepoints were excluded to ensure data quality.
